# First Complete *Squash leaf curl China virus* Genomic Segment DNA-A Sequence from East Timor

**DOI:** 10.1128/genomeA.00483-17

**Published:** 2017-06-15

**Authors:** Solomon Maina, Owain R. Edwards, Luis de Almeida, Abel Ximenes, Roger A. C. Jones

**Affiliations:** a School of Agriculture and Environment, Faculty of Science, the University of Western Australia, Crawley, Western Australia, Australia; b Institute of Agriculture, Faculty of Science, the University of Western Australia, Crawley, Western Australia, Australia; c Cooperative Research Centre for Plant Biosecurity, Canberra, Australian Capital Territory, Australia; d CSIRO Land & Water, Floreat Park, Western Australia, Australia; e Seeds of Life Project, Ministry Agriculture and Fisheries, Dili, East Timor; f DNQB-Plant Quarantine International Airport Nicolau Lobato Comoro, Dili, East Timor; g Department of Agriculture and Food Western Australia, South Perth, Western Australia, Australia

## Abstract

We present here the first complete *Squash leaf curl China virus* (SLCCV) genomic segment DNA-A sequence from East Timor. It was isolated from a pumpkin plant. When compared with 15 complete SLCCV DNA-A genome sequences from other world regions, it most resembled the Malaysian isolate MC1 sequence.

## GENOME ANNOUNCEMENT

As part of a project to examine genomic connectivity between viruses infecting crops in northern Australia and nearby southeast Asian countries, virus genomes from plant samples from East Timor and Australia were compared (1–10). In 2015, 15 and 22 cucurbit leaf samples with virus-like symptoms were collected in four locations in East Timor and Broome in northwest Australia, respectively, and subjected to next generation sequencing. A complete genomic segment *Squash leaf curl China virus* (SLCCV) DNA-A sequence was obtained from pumpkin sample TM4 from Dili in East Timor. SLCCV belongs to the *Begomovirus* genus, in the family *Geminiviridae*. Members of this genus have two circular single stranded DNA (ssDNA) components (DNA-A and DNA-B) and a genome of approximately 2.7 kb encapsidated within twinned icosahedral particles (11–14). SLCCV is whitefly transmitted and has a restricted host range within the *Cucurbitaceae* family, infecting squash and pumpkin, but not melon or cucumber (15). It occurs in many world regions including parts of southeast Asia (13–15), but has not been found previously in East Timor or Australia. RNA-Seq with rRNA-plant depletion provides reliable metagenomic detection of polyadenylated and nonpolyadenylated RNA viruses and can also detect DNA viruses (16–18). This approach detected SLCCV in sample TM4 (designated isolate SLCCV T4-D).

The 15 East Timorese samples were blotted onto fast technology for analysis of nucleic acids (FTA) cards (19) before dispatch to Australia. The Australian samples studied were recently collected leaves. Total RNA was extracted from both sample types using a ZR Plant RNA MiniPrep kit (Zymo Research). The total RNA extracts were treated with RNase-free DNase (Invitrogen). Quality control was done and RNA subjected to total RNA sample preparation Ribo-Zero plant kit (catalogue no. RS-122-2401, Illumina) as described previously (1–10). Sequencing was by HiSeq 2500 using a Truseq SBS KIT (Illumina) with 151 cycles to generate paired-end reads in a multiplex of 24 samples in one lane. Reads were assembled and genomes annotated using CLC Genomics Workbench 6.5 (CLC bio) and Geneious 8.1.7 (Biomatters) (20, 21).

FTA card sample TM4 yielded 12,867,884 reads and, after trimming, 12,244,994 remained. *De novo* assembly generated 250 contigs and 75,817 reads mapped to the contig of interest with coverage of 4,119. Final coding genome length was 2,737 nucleotides (nt). As with other begomoviruses, SLCCV coded for a pre-coat protein, protein C4, and replication-associated protein C1. A BLAST-based search with pairwise sequence comparison (PASC) tool (22), revealed the T4-D genome sequence most resembled Malaysian isolate MC1, GenBank accession number EF197940, with 95.0% nt identity. Since no SLCCV was detected in any Australian samples, further sampling is needed to establish whether SLCCV has spread to Australia from nearby southeast Asian countries. Comparison of any Australian genomic sequences found with ones from neighboring countries would be required.

### Accession number(s).

This sequence was deposited at DDBJ/EMBL/GenBank under accession number KY652743.
